# Effects of motion paradigm on human perception of tilt and translation

**DOI:** 10.1038/s41598-022-05483-6

**Published:** 2022-01-26

**Authors:** Gilles Clément, Kara H. Beaton, Millard F. Reschke, Scott J. Wood

**Affiliations:** 1 Lyon Neuroscience Research Center, CNRS UMR5292-INSERM U1028-University of Lyon, Impact Team, 16 Avenue Doyen Lepine, 69676 Bron Cedex, France; 2 The Aerospace Corporation, El Segundo, CA USA; 3 NASA Neuroscience Laboratory, Johnson Space Center, Houston, TX USA

**Keywords:** Neuroscience, Sensory processing, Physiology, Neurophysiology

## Abstract

The effect of varying sinusoidal linear acceleration on perception of human motion was examined using 4 motion paradigms: off-vertical axis rotation, variable radius centrifugation, linear lateral translation, and rotation about an earth-horizontal axis. The motion profiles for each paradigm included 6 frequencies (0.01–0.6 Hz) and 5 tilt amplitudes (5°–20°). Subjects verbally reported the perceived angle of their whole-body tilt and the peak-to-peak translation of their head in space and used a joystick capable of recording 2-axis motion in the sagittal and transversal planes to indicate the phase between the perceived and actual motions. The amplitudes of perceived tilt and translation were expressed in terms of gain, i.e., the ratio of perceived tilt to equivalent tilt angle, and the ratio of perceived translation to equivalent linear displacement. Tilt perception gain decreased, whereas translation perception gain increased, with increasing frequency. During off-vertical axis rotation, the phase of tilt perception and of translation perception did not vary across stimulus frequencies. These motion paradigms elicited similar responses in roll tilt and interaural perception of translation, with differences likely due to the influence of naso-occipital linear accelerations and input to the semicircular canals that varied across motion paradigms.

## Introduction

Head movements in the sagittal (pitch) and transversal (roll) planes elicit signals from the vestibular system, which are expressed through eye movements and perceptions of self-motion. Traditionally, eye movements are evaluated to assess vestibular function; numerous studies have investigated how linear and angular acceleration affects ocular motility^1^. After the pioneering work of Ernst Mach in 1875 (translated by Ref.^2^), perception of self-motion during angular acceleration was extensively studied, but much less is known on the perception of self-motion during linear acceleration.

The greatest challenge for the human navigation system stems from the inherent ambiguity between linear acceleration due to a gravitational field (e.g., tilt) and linear acceleration due to inertial motion (e.g., translation). This tilt-translation enigma, first postulated by Einstein^3^, is demonstrated in the intrinsic inability of otolith afferents to adequately distinguish these linear accelerations. The linear transducers of the inner ear require the central nervous system (CNS) to discriminate tilt and translation, and to delineate the appropriate compensatory reflexes. In general, the CNS correctly interprets natural movements during everyday life; however, this ability is compromised in patients with a dysfunctioning vestibular system^4^ and in individuals working in complex 3-dimensional environments, such as the neutrally buoyant surroundings experienced by deep sea divers^5,6^ and the altered acceleration fields faced by aviators^7,8^ and astronauts^9–13^.

Two theories have been proposed on how neural strategies resolve this tilt-translation ambiguity: (1) frequency segregation, in which low frequency motion is interpreted as tilt, and high frequency motion is interpreted as translation^14–16^; and (2) integration, in which the brain uses information from other sensory sources, such as the semicircular canals, vision, or proprioception, to distinguish between the 2 types of motion^17–23^. These studies provided evidence that frequency segregation and multisensory integration both play a role in determining ambiguous motions.

**Figure 1:**
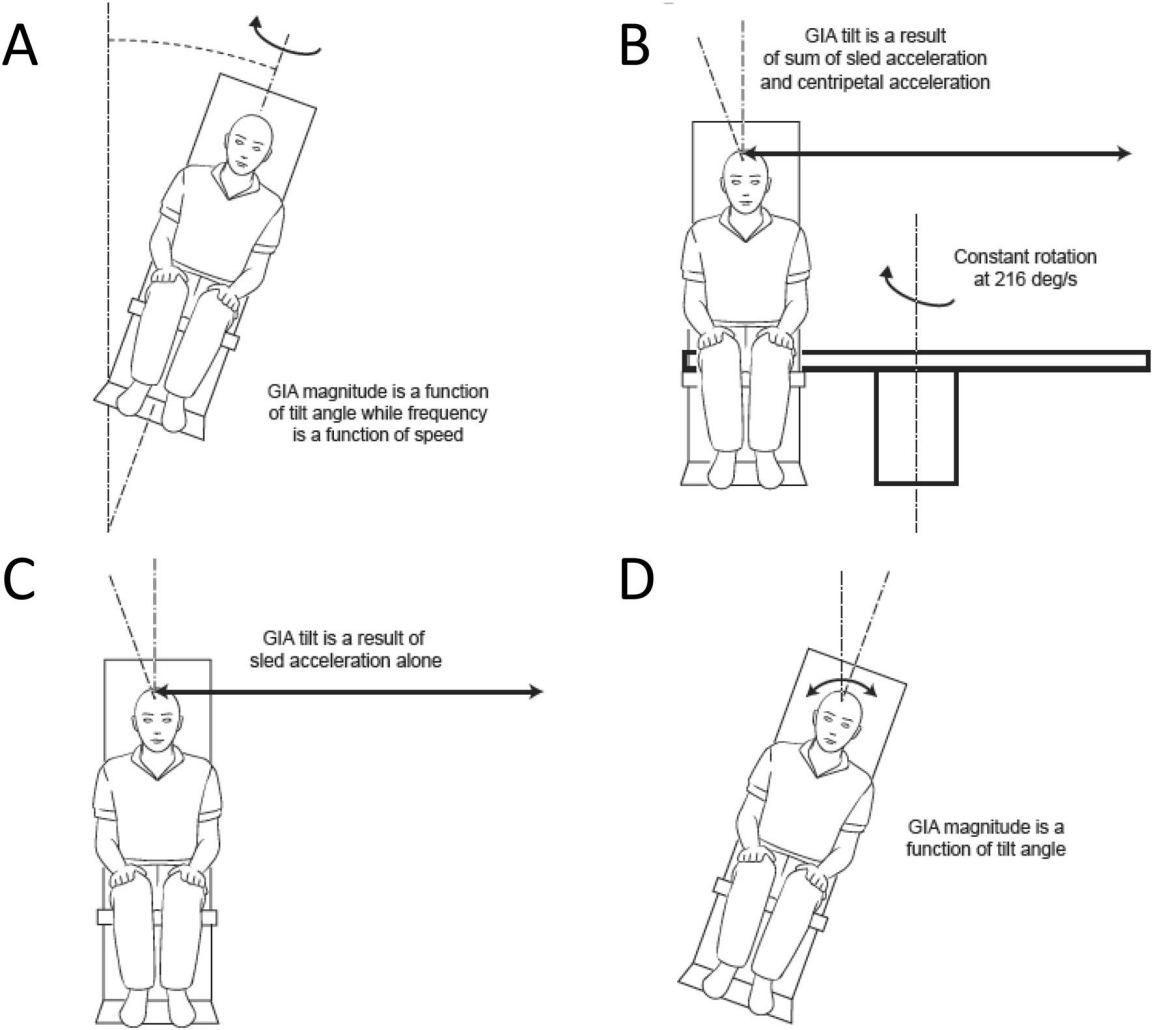
The 4 motion paradigms used in this study. (**A**) Off-vertical axis rotation (OVAR) generated linear acceleration along both the interaural axis and the naso-occipital axis without input to the semicircular canals. (**B**) Variable radius centrifuge (VRC) generated both a centripetal acceleration along the interaural axis and a Coriolis linear acceleration along the naso-occipital axis without input to the semicircular canals. (**C**) Linear lateral acceleration (LLT) generated linear acceleration along the interaural axis at low frequency (due to the limited sled length without naso-occipital linear acceleration and without input to the semicircular canals. (**D**) Earth horizontal axis rotation (EHAR) generated a linear acceleration along the interaural axis without naso-occipital linear acceleration, and input to the semicircular canals declined at low frequency of rotation.

We examined vestibular perception elicited by 4 different motion paradigms, each providing subtly distinct linear acceleration stimuli to the inner ear. To maintain consistency across motion platforms, peak acceleration, frequency, and tilt amplitude stimuli remained constant across each paradigm. Variations in stimuli across motion platforms arose by changing the direction and magnitude of the resultant linear acceleration. Off-vertical axis rotation (OVAR) employed constant velocity rotation (thereby minimizing the angular cues sensed by the semicircular canals^17^) about an axis tilted with respect to gravity, creating a dynamic linear acceleration stimulus equal to the sine function of the tilt angle (Fig. 1A). During OVAR, the linear acceleration stimulus was equally present in both the pitch and roll planes. Variable radius centrifugation (VRC) also employed constant velocity rotation (i.e., concordant tilt cues from the semicircular canals were absent); however, linear acceleration resulted from the centripetal acceleration in the transversal plane, which was a function of the rotational velocity and the square of distance from the axis of rotation, and Coriolis acceleration in the sagittal plane, which was a function of the linear velocity and oscillation frequency (Fig. 1B). In addition, both the centripetal and Coriolis accelerations were combined with the gravito-inertial acceleration. Both OVAR and VRC involve angular acceleration to a constant velocity, with the decay of the canal-mediated per-rotational response determined by velocity storage mechanisms^24,25^. Linear lateral translation (LLT) involved linear acceleration in the transversal plane, which also produced directional changes in the resultant acceleration vector itself (Fig. 1C). Rotation about an Earth horizontal axis (EHAR) was induced by tilt about the naso-occipital axis (Fig. 1D). EHAR was the only paradigm that induced concordant cues from both the semicircular canals and the otoliths, rendering this paradigm most similar to natural head movements.

Past studies have assumed equivalence among these four motion paradigms that provide comparable resultant stimuli. For example, Glasauer^26^ examined how frequency affects the “hilltop illusion” using a variable radius centrifuge to test low frequencies, and a linear sled to test high frequencies. Similarly, Merfeld et al.^27^ investigated how frequency affects motion perception and eye movements using variable radius centrifugation to test low frequency profiles, and linear translation to test high frequency profiles. Investigators often use particular motion platforms because hardware is limited in their laboratory: most laboratories traditionally house only one or 2 devices, and each device is subject to its own limitations. For instance, linear sleds are valuable for providing single axis accelerated motion but require excessively long track lengths for studying lower frequency motions. Although centrifuges allow testing at lower frequencies, they require high mechanical torques during high rates of rotation, and single-axis perceptions are complicated by Coriolis acceleration. Nonetheless, it is reasonable to question whether the CNS interprets actual tilt and actual translation differently than concomitant tilt and translation, depending on the mode in which the stimuli are delivered to the body. Our study is unique because it specifically evaluates the issue of equivalence across 4 motion devices.

The current study specifically addressed the following 3 questions: (1) Does the frequency and the amplitude of tilt determine if the motion is perceived as tilt or as translation? (2) Is there a crossover frequency range where perception of tilt and translation motion are roughly equal? (3) How do the previous 2 questions vary among the 4 motion paradigms? We predicted that both the frequency and the amplitude of tilt would determine if the motion was perceived as tilt or as translation, as proposed by the frequency segregation hypothesis. We anticipated that the amplitude of perceived tilt would decrease and the amplitude of perceived translation would increase with increasing frequency, but that tilt perception would be stronger for the OVAR and EHAR paradigms, where actual tilt was involved, and that translation perception would be stronger for the VRC and LLT paradigms, where actual translation was involved. We also hypothesized that perception of either tilt or of translation would vary as a function of the tilt amplitude stimulus at a given frequency. We also assumed, based on the results of previous perception studies using OVAR^28,29^, that the phase of perception of either tilt or translation motion would be independent of both the frequency and the amplitude of tilt. While we previously reported a crossover frequency around 0.3 Hz during OVAR^30^, another objective was to examine how consistent this crossover frequency was across the different motion paradigms.

## Methods

### Participants

Twelve healthy subjects (8 male, 4 female; ranging in age from 25 to 49 years; mean 34.6 years) participated in this study. All subjects passed a modified United States Air Force Class III medical examination and had no known history of vestibular or oculomotor abnormalities. The test procedures were approved by the NASA Johnson Space Center Institutional Review Board and were performed in accordance with the ethical standards laid down in the 1964 Declaration of Helsinki. All subjects gave a written informed consent before participating in the study. All subjects completed the study.

### Motion stimuli

All tests were performed in the Neuroscience Laboratory at NASA Johnson Space Center. The devices used for OVAR, VRC, LLT, and EHAR have been described in previous publications. OVAR was performed on NASA’s off-vertical axis rotator^30^, VRC was performed on NASA’s variable radius centrifuge^13^, and both LLT and EHAR were performed on NASA’s tilt-translation sled^13^.

The off-vertical axis rotator and variable radius centrifuge devices used a direct-drive DC motor and a high precision tachometer-based servo controller (Neuro Kinetics Inc., Pittsburgh, PA) to control motion and maintain stability during rotation. The off-vertical axis rotator incorporated a tilt stand (Intelligent Lift Systems, Galveston, TX) coupled to an electromechanical linear actuator (Super-Pac, Duff-Norton, Charlotte, NC) to tilt the axis of rotation between upright and 20° off-vertical. In addition to velocity feedback from the tachometer, a position encoder provided an alternate source of information about the chair motion. A linear screw drive mechanism in the variable radius centrifuge translated the chair platform up to 25 cm on either side of center, and a position encoder provided real-time feedback of chair radius. The tilt-translation sled generated translational motion along a 4-m air bearing track via 3 linear motors operated in series in a single magnet track. A precision linear encoder provided feedback that was used to control translation motion and maintain stability. The tilt motion was provided by dual-wheel friction wheels with direct drive servo motors and a pivoting yoke assembly providing the dynamic displacement.

**Figure 2:**
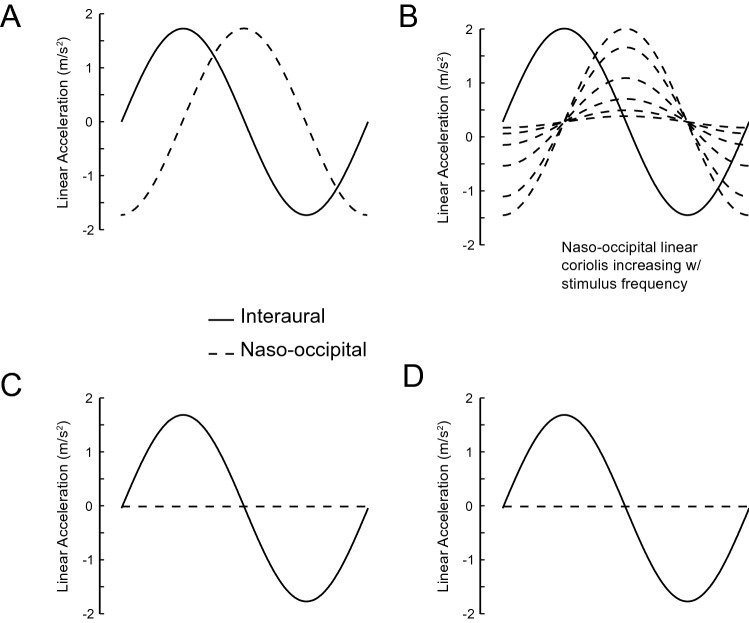
Modulations of linear acceleration along the interaural axis (solid) and the naso-occipital axis (dashed) during the 4 motion paradigms generating a 10° tilt magnitude. (**A**) Off-vertical axis rotation (OVAR) was consistent across all frequencies (velocities). (**B**) Variable radius centrifuge (VRC) generated a Coriolis linear acceleration that increased with stimulus frequency. (**C**) Linear lateral acceleration (LLT) generated pure linear acceleration along the interaural axis with no naso-occipital linear acceleration. (**D**) Earth horizontal axis rotation (EHAR) generated a linear acceleration along the interaural axis due to roll tilt relative to gravity with no naso-occipital linear acceleration.

The stimulus frequencies and tilt angles used during each paradigm are defined in Table 1. Due to the mechanical limits of the variable radius centrifuge and the tilt-translation sled, some profiles did not exactly match those used in other paradigms, and some profiles could not be tested at all. However, the peak acceleration stimulus that defined each tilt amplitude remained constant across paradigms. Figure 2 and Table 2 describe the differences between the stimuli across paradigms.

**Table 1:** Stimulus frequency and actual or simulated tilt angles used for each motion paradigm. *OVAR* off-vertical axis rotation (actual tilt), *VRC* variable radius centrifuge (simulated tilt), *LLT* linear-lateral translation (simulated tilt), *EHAR* Earth-horizontal axis rotation (actual tilt). NA indicates when motion was not possible due to device limitations.

Frequency	OVAR	VRC	LLT	EHAR
0.01875 Hz	10°	10°	0.2°	10°
0.0375 Hz	10°	10°	0.6°	10°
0.075 Hz	10°	10°	2.5°	10°
0.15 Hz	5°	5°	5°	5°
	10°	10°	10°	10°
	15°	15°	NA	15°
	20°	20°	NA	20°
0.3 Hz	10°	10°	10°	10°
0.6 Hz	5°	5°	5°	5°
	10°	10°	10°	10°
	15°	NA	15°	NA
	20°	NA	NA	NA

**Table 2:** Stimulus parameters for the various motion paradigms at each frequency. *F* frequency, *a* linear acceleration, *v* linear velocity, *d* peak-to-peak amplitude, *θ* tilt angle.

f (Hz)	OVAR/EHAR							
	a (m/s^2^)	v (m/s)	d (m)	θ (°)				
0.01875	1.70	14.46	122.74	10				
0.0375	1.70	7.23	30.68	10				
0.075	1.70	3.61	7.67	10				
0.15	1.70	1.81	1.92	10				
0.3	1.70	0.90	0.48	10				
0.6	1.70	0.45	0.12	10				

f (Hz)	VRC				LLT			
	a (m/s^2^)	v (m/s)	d (m)	θ (°)	a (m/s^2^)	v (m/s)	d (m)	θ (°)
0.01875	1.73	14.68	124.63	10	0.03	0.23	1.95	0.2
0.0375	1.73	7.34	31.16	10	0.11	0.46	1.95	0.6
0.075	1.73	3.67	7.79	10	0.43	0.93	1.95	2.5
0.15	1.73	1.84	1.95	10	1.73			
0.3	1.73	0.92	0.49	10	1.73			
0.6	1.73	0.46	0.12	10	1.73			

During OVAR, subjects were accelerated in the upright orientation about their longitudinal axis at 10°/s^2^ to the desired frequency. Once the sensation of rotation ceased (after approximately 2 min), subjects were tilted to the desired angle at 4°/s and held in this position while continuing to rotate about their longitudinal axis. Subjects were always moved to the upright position prior to any frequency changes to help mitigate symptoms of motion sickness.

During VRC, subjects were accelerated about their longitudinal axis at 10°/s^2^ to a rotational velocity of 216°/s. Once the sensation of rotation ceased (after approximately 2 min), subjects remained in the upright position and were oscillated laterally at the prescribed frequencies. Oscillation amplitudes were selected such that the maximum resultant roll-tilt stimulus equaled the desired tilt angle. VRC was performed at a rotational velocity of 216°/s so that the linear acceleration stimulus provided during 0.6 Hz VRC matched that of 0.6 Hz OVAR.

During LLT, subjects remained in the upright position and were oscillated laterally at the defined frequencies. Again, oscillation amplitudes were selected such that the maximum resultant roll-tilt equaled the desired tilt angle. Track length limited these tilt stimuli at the lower frequencies, and therefore the 0.01875 Hz, 0.0375 Hz, and 0.075 Hz profiles only produced maximum roll tilts of 0.2°, 0.6°, and 2.5°, respectively.

During EHAR, subjects were rotated about the nasal-occipital axis at rotation rates defined by the different frequencies and tilt amplitudes defined by the desired tilt angles. A vertical linear actuator below the seat allowed the subject to be raised and lowered to visually align the external auditory canal with the rotation axis.

### Experimental protocol

During each test, subjects were restrained in an upright seated position with their head oriented in the naturally erect position. Head position was maintained via adjustable foam pads positioned around the forehead and base of the neck. A 4- or 5-point harness restrained the shoulders and torso, and additional straps provided restraint to the shins and feet. When necessary, formable Vac-Pacs^®^ (Olympic Medical, Seattle, WA) immobilized the mid-torso and uniformly distributed pressure. Noise reduction headphones provided 2-way audio communications and helped minimize extraneous orientation cues. Subjects wore light occluding goggles to ensure that testing was performed in complete darkness. Joystick movements and motion feedback signals were simultaneously sampled at 100 Hz using custom LabWindows software that also controlled the stimulus profiles.

Each subject was tested on all 4 motion paradigms, and testing on each device occurred on separate days. The order of devices, stimulus frequency, tilt amplitude, and perception reporting (tilt or translation first), were partially counterbalanced using a Latin Square design across subjects to minimize test order effects. Testing was stopped with the onset of slight nausea. Subjects who developed symptoms were tested again on that device during a subsequent session.

During each motion profile, subjects verbally reported the amplitude of the perceived tilt (peak displacement) and the amplitude of the perceived translation (peak-to-peak displacement) of their head in space. When subjects perceived tilt, they were asked to report where was the axis of rotation (e.g., above the head, head level, body level, below the body). Subjects were shown visual diagrams and movies depicting various scenarios of tilt, translation, or combinations thereof. These diagrams and movies are provided as Supplementary Information. Tilt was defined as the maximum number of degrees from the upright vertical, and translation was defined as the peak-to-peak displacement of the head in space. Subjects were free to report displacement in either metric or imperial units as these could be easily converted, although most reported in imperial. While the verbal reports along do not allow direct phase measures, the magnitudes are reported in real-world units that are meaningful to the subjects without the need for normalizing or scaling the outputs as required for joystick or similar objective somatosensory measures.

During OVAR, a joystick capable of recording 2-axis motion in the sagittal and transversal planes was also used to define the subjects’ perceived directions of tilt and translation displacement in real time and to calculate phase between the motion stimulus and perception^27^. At the beginning of each test session, definitions of tilt and translation and instructions on proper use of the joystick were reinforced to ensure consistency across test sessions and among subjects. During tilt (translation), subjects moved the joystick forward when they felt pitched down (head moving forward), left when they felt left ear down (head moving left), back when they felt pitched up (head moving backwards), and right when they felt right ear down (head moving right). Throughout perception reporting, subjects focused on sensations of tilt and translation separately to account for potential differences in phase between the 2 perceptions.

### Data analysis

Joystick and motion stimuli data were filtered with median hybrid filter^31^. Nonlinear least squares sinusoidal curve fits were used to characterize the phase of the modulation of joystick responses relative to the varying sinusoidal linear acceleration stimulus. Curve fits were performed on successive cycles and averaged for each condition. An example of a raw tracing and curve fit is provided as Supplementary Information. Phases were determined for pitch and roll tilt and the medial–lateral and anterior–posterior head translation, with positive leading and negative lagging. A minimum of 4 cycles of tilt joystick data and 4 cycles of translation joystick data were collected for each stimulus condition.

When comparing the amplitude of tilt and perceptions of translation across frequency, subject verbal reports of perceived tilt (in deg) and perceived translation (in displacement) were converted to gain, i.e., the ratio of perceived tilt angle to equivalent tilt angle, or the ratio of perceived translation to equivalent linear displacement. Equivalent tilt angle was the amount of tilt the subject would theoretically perceive if the linear acceleration stimulus was interpreted solely as tilt, and equivalent linear displacement was the amount of displacement the subject would theoretically perceive if the linear acceleration stimulus was interpreted solely as translation. The equivalent tilt angle during OVAR, VRC, and EHAR equaled the actual tilt stimulus, and the equivalent linear displacement during LLT equaled the actual lateral displacement. Values for equivalent tilt angles, θ, and equivalent linear displacements, d, are given in Tables 2 and 3. Note these are derived mathematically from the primary stimulus parameters controlled for each motion profile, e.g. frequency and tilt angle for OVAR and frequency and linear displacement for LLT. This results in gain calculations being assessed against a perfect double-integration of acceleration sensed by the otolith organs. For example, the equivalent translation at 0.01875 Hz ranges between 123 and 125 m for OVAR, EHAR and VRC (see Table 2). We acknowledge that this results in inherently small translation gains at lower frequencies for these paradigms.

**Table 3:** Stimulus parameters for the various motion paradigms at each tilt angle. *F* frequency, *a* linear acceleration, *v* linear velocity, *d* peak-to-peak amplitude, *θ* tilt angle.

f (Hz)	θ (°)	OVAR			EHAR		
		a (m/s^2^)	v (m/s)	d (m)	a (m/s^2^)	v (m/s)	d (m)
0.15	5	0.85	0.91	0.96	0.85	0.91	0.96
	10	1.70	1.81	1.92	1.70	1.81	1.92
	15	2.54	2.69	2.86	2.54	2.69	2.86
	20	3.36	3.56	3.78	3.36	3.56	3.78
0.6	5	0.85	0.23	0.06	0.85	0.23	0.06
	10	1.70	0.45	0.12	1.70	0.45	0.12
	15	2.54	0.67	0.18			
	20	3.36	0.89	0.24			

f (Hz)	θ (°)	VRC			LLT		
		a (m/s^2^)	v (m/s)	d (m)	a (m/s^2^)	v (m/s)	d (m)
0.15	5	0.86	0.91	0.97	0.86	0.91	0.97
	10	1.73	1.84	1.95	1.73	1.84	1.95
	15	2.63	2.79	2.96			
	20	3.57	3.79	4.02			
0.6	5	0.86	0.23	0.06	0.86	0.23	0.06
	10	1.73	0.46	0.12	1.73	0.46	0.12
	15				2.63	0.7	0.18
	20						

When comparing verbal reports of tilt and translation across tilt angles (i.e., when the frequency was held constant at 0.15 Hz and 0.6 Hz), the perception of the amplitudes of tilt or translation were expressed in terms of their raw values. This allowed for direct examination of potential linearity effects across increasing tilt angle, and for general comparisons between the magnitudes of tilt and translation at lower (e.g., 0.15 Hz) and higher (e.g., 0.6 Hz) frequencies.

### Statistical analysis

The normality of distribution of the variables was determined using the Shapiro–Wilk Test. Variables that were not normally distributed (VRC tilt gain, VRC perceived tilt) were log10 transformed. The log10 transformed values yielded a dataset that more closely conforms to a normal distribution. A repeated-measures analysis of variance (ANOVA) with 2 factors (frequency: 0.01875 Hz, 0.0375 Hz, 0.075 Hz, 0.15 Hz, 0.3 Hz, 0.6 Hz; and motion stimuli: OVAR, VRC, LLT, EHAR) was used to detect differences between tilt and translation gain across motion paradigms. A repeated-measures ANOVA with 2 factors (stimulus angle: 5°, 10°, 15°, 20°; and motion stimuli: OVAR, VRC, LLT, EHAR) was used to detect differences between the amplitude of perceived tilt and perceived translation across motion paradigms. Bonferroni correction was used to adjust for multiple comparisons.

## Results

### Perception of roll tilt and lateral translation

**Figure 3:**
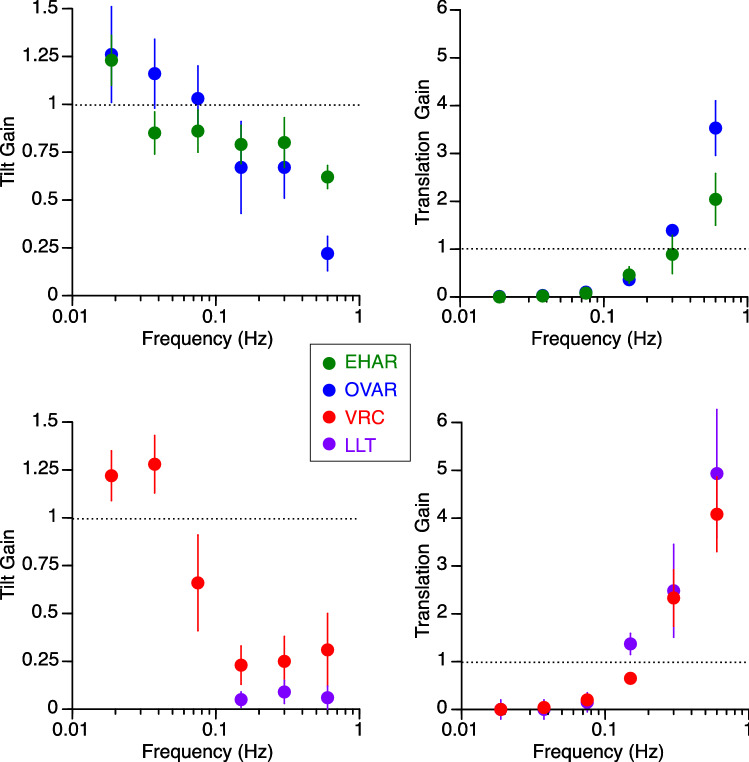
Mean ± standard error of tilt gain and translation gain (from 12 subjects) as a function of frequency during each of the 4 motion paradigms generating an equivalent tilt of 10°. Tilt gain is the ratio of perceived tilt angle and equivalent (i.e., the actual or the resultant tilt from linear acceleration and gravitation acceleration stimuli) tilt angle. Translation gain is the ratio of perceived translation displacement and the equivalent linear displacement.

Frequency had a significant effect on tilt gain and on translation gain (Fig. 3). A 2-factor repeated measures ANOVA indicated that this effect was observed for all 6 frequencies during OVAR, VRC, and EHAR for tilt gain (F(5,215) = 13.28, *p* < 0.001) and for translation gain (F(5,215) = 73.81, *p* < 0.001). The effect of frequency on translation gain was also significant during all motion paradigms at frequencies of 0.15 Hz, 0.3 Hz, and 0.6 Hz (F(2,143) = 24.28, *p* < 0.001), although significant differences were determined across motion paradigms (F(3,143) = 4.77, *p* < 0.001). At 10° of tilt, tilt gains at the lower frequencies (< 0.15 Hz) were greatest during OVAR and EHAR, the paradigms involving actual rotation, whereas translation gains at the higher frequencies were greatest during LLT and VRC, the paradigms incorporating actual translation. Figure 3 includes LLT results at stimulus tilt amplitudes of 0.2°, 0.6°, and 2.5° for the 3 lowest frequencies. Although these data points are not completely compatible with the other 10° tilt paradigms, their mean values follow the same trend of minimal translation gain. However, high standard errors exist that are attributed to the difficulty in perceiving any translation at lower stimulus frequencies.

**Figure 4:**
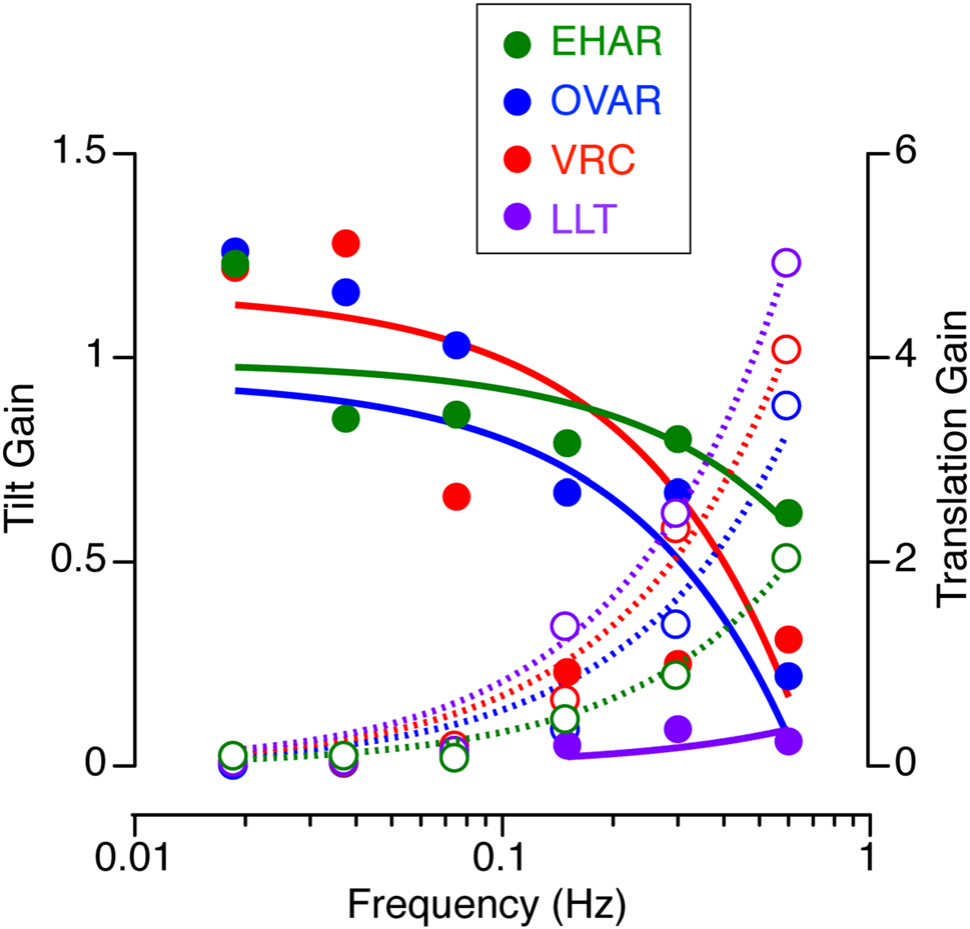
Data from Fig. 3 superimposed in a single diagram. The closed symbols and the solid fit lines show the tilt gains across all motion paradigms. The open symbols and the dashed fit lines show the translation gain across all motion paradigms. Curve fittings are linear curve fits. The crossover frequency between tilt gain and translation gain occurred between 0.15 Hz and 0.6 Hz depending on the motion paradigms.

Since the translation gains use linear displacements derived from double integration of equivalent acceleration for OVAR, VRC and EHAR, these gains would be inherently small due to perceptual tendency to underestimate the relatively large displacements (123–125 m, Table 2). It is important to note that the actual translations reported were in fact quite small at these frequencies, e.g. 0.88 ± 0.47 m, 0.36 ± 0.52 m, and 0.15 ± 0.35 m (mean ± std) for OVAR, VRC and EHAR, respectively. Based on linear fits across frequencies, the crossover frequency range between the gain of perceptions of tilt and the gain of perceptions of translation appears to occur at < 0.15 Hz for LLT, 0.3 Hz for OVAR and VRC, and 0.6 Hz for EHAR (Fig. 4). The trend in the cut-off frequency across conditions was not tested statistically.

**Figure 5:**
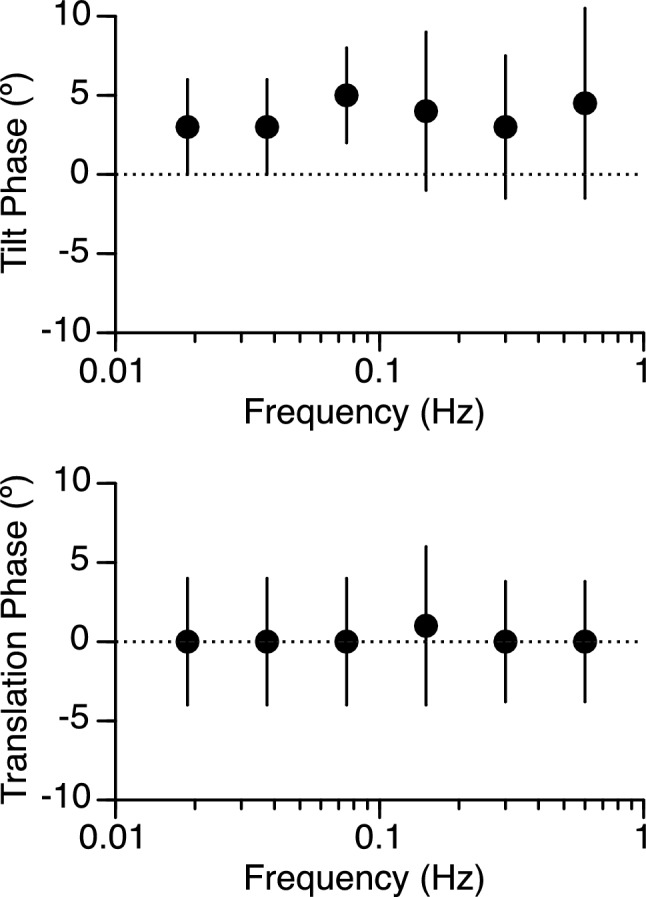
Mean ± standard error of the phase of the joystick tilts and translation displacements as a function of motion frequency during off-vertical axis rotation (OVAR) in 12 subjects.

Perception of tilt lead the OVAR stimulus by 3.8° ± 0.88° (mean ± SD), whereas perceived translation remained in phase across the 6 stimulus frequencies (Fig. 5). Neither the perception of tilt nor the perception of translation were significantly altered by stimulus frequency during OVAR. Note this phase data was not available for the three other motion paradigms.

Table 4 summarizes the number of subjects who perceived the axis of rotation during tilt motion at their head and the number of subjects who perceived this axis at another point along their long-body axis. During OVAR and VRC most subjects reported that the axis of tilt motion was below their head, and the number of subjects who reported this increased as the frequency increased. The fact that the axis of rotation was perceived below the head could account for the larger perceived translation of the head at high frequencies. During EHAR, most subjects perceived the rotation axis to be at their head (which was correct), or below their head (e.g., during rotation at 0.15 Hz). Only a few subjects were able to report the location of the rotation axis during LLT because of the small amplitude of perceived tilt in this paradigm.

**Table 4:** Number of times subjects perceived body tilt with the axis of rotation above, at, or below their head for each frequency during the various motion paradigms. ^a^Frequency includes all tilt angles tested (see Table 3).

Frequency	Axis of Rotation	OVAR	VRC	LLT	EHAR
0.01875 Hz	Above head	0	1	0	2
	At head	0	1	1	4
	Below head	11	10	0	5
	Not reported	1	0	11	1
0.0375 Hz	Above head	0	0	0	1
	At head	0	2	0	7
	Below head	12	10	0	4
	Not reported	0	0	12	0
0.75 Hz	Above head	0	2	0	1
	At head	0	0	0	8
	Below head	11	6	1	3
	Not reported	1	4	11	0
0.15 Hz^a^	Above head	0	0	0	6
	At head	2	4	0	19
	Below head	36	21	3	22
	Not reported	10	24	21	1
0.3 Hz	Above head	0	1	1	1
	At head	0	0	1	6
	Below head	9	4	0	5
	Not reported	3	7	10	0
0.6 Hz^a^	Above head	0	1	0	1
	At head	0	1	2	16
	Below head	27	4	2	7
	Not reported	21	18	32	0

**Figure 6:**
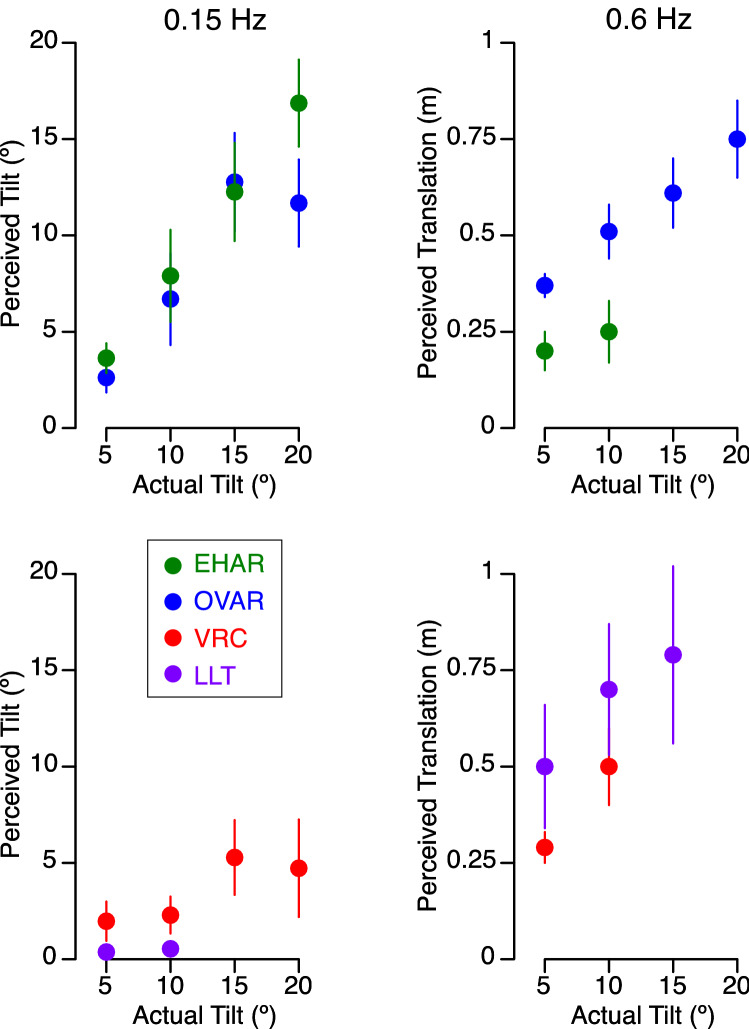
Mean ± standard error of perceived amplitudes of tilt and perceived amplitudes of translation as a function of stimulus tilt angle during the 4 motion paradigms at 0.15 Hz and at 0.6 Hz. Refer to Table 1 for the clarification of trials conducted at each angle and frequency.

The angle of the perceived tilt at 0.15 Hz increased with increasing tilt stimulus (Fig. 6). A 2-factor repeated measures ANOVA indicated that this effect was significant for all 4 angles of tilt during OVAR, VRC, and EHAR (F(3,143) = 14.16, *p* < 0.001), but significant differences were observed across motion paradigms (F(2,143) = 14.49, *p* < 0.01). This increase was most clearly observed during the EHAR and OVAR, where actual tilt was involved, than during the VRC and LLT. Additionally, responses during EHAR were more linear than those given during OVAR, possibly because the actual stimulus provided during EHAR was tilt in a single dimension, whereas during OVAR, subjects tended to perceive conical motion with translation of their head in space (see Table 4).

The amplitude of the perceived translation at 0.6 Hz also increased with increasing tilt stimulus (Fig. 6). A 2-factor repeated measures ANOVA indicated that this effect was significant for 5° and 10° tilt for OVAR, LLT, and VRC (F(1,71) = 4.14, *p* = 0.04). The amplitude of perceived translation was the lowest during EHAR, where the acceleration stimulus at this frequency was most consistently interpreted as tilt.

### Perception of pitch tilt and fore-aft translation

During LLT and EHAR the linear acceleration stimulus was provided solely in the roll plane and no subject reported motion in any other plane during these 2 paradigms. However, perceptions of tilt in pitch occurred during OVAR and VRC, the 2 paradigms where the linear stimulus acted in both the roll and the pitch planes. No subject reported asymmetries between the pitch and the roll planes during any of the OVAR paradigms. However, asymmetries did exist during VRC, presumably as a result of Coriolis linear acceleration. Although no pitch tilt was reported during VRC, half of the subjects (6 out of 12) perceived a quantifiable amount of fore-aft translational motion at frequencies greater than 0.15 Hz. However, although the 0.6 Hz VRC profile was designed to match the 0.6 Hz OVAR stimulus, no subject reported equivalent amounts of translation in the pitch and the roll planes during these 2 paradigms. The primary reason why tilt in the pitch plane was not perceived was because Coriolis acceleration was strongest at the highest frequencies when the angle of perceived tilt in general tended to be small.

## Discussion

Three questions were examined in hopes of providing a more complete picture of how the CNS interprets ambiguous motion cues: (1) Do perception of tilt and translation motion depend on frequency and amplitude of tilt? (2) Is there a crossover frequency range where perception of tilt and perception of translation motion are roughly equal? (3) How do the previous 2 questions vary among the 4 motion paradigms? The main finding that similar responses in roll tilt and interaural perception of translation are elicited by these different motion profiles is important in that it confirms the extent to which findings from other studies in the literature can be integrated.

### Effects of stimulus amplitude and frequency

During a roll tilt paradigm where the axis of rotation could be systematically varied, Rader et al.^32^ concluded that magnitude estimates of tilt perception were influenced by knowledge of swing geometry. Using similar reasoning we hypothesized that motion perceptions would vary across platforms that involve actual tilt or actual translation, depending on how the subject interpreted the linear stimulus. OVAR and EHAR involved actual whole-body tilt with respect to gravity, whereas the swinging gravity vector produced during VRC and LLT generated the equivalent of a tilt, which arose from tilting the gravito-inertial acceleration (GIA) vector relative to gravity. Likewise, VRC and LLT involved actual lateral translation, whereas the perception of translation experienced during OVAR and EHAR resulted from the varying orientation of the acceleration vector. Consequently, the amplitude of perceived tilt was larger during OVAR and EHAR, whereas the amplitude of perceived translation was larger during VRC and LLT (Fig. 6).

The results of this study confirm that the amplitude of perceived tilt increased with the actual or equivalent tilt angle of the stimulus^13,30,33^. Using static roll tilt, Müller^34^ observed that subjects tend to overestimate the angle of tilts less than 60° and underestimate the angle of larger tilts. Although dynamic tilt was used in our study, tilt gains higher than unity were observed during EHAR, OVAR, and VRC for equivalent tilt angles of 10°, indicating that subjects overestimated the angle of tilt at low frequency (< 0.04 Hz). The differences in tilt gain across the 4 motion paradigms could be because the linear acceleration component due to gravity along the otolith macula during EHAR changed as a function of tilt, whereas during OVAR the linear acceleration was constant and just changed in direction over time. During VRC, the centripetal acceleration was not constant; it was greatest at the maximum velocity (which was at 0 m off-center), whereas the radial acceleration was the greatest at the largest distance. There were no significant intra-individual findings, e.g., individuals tending to favor tilt across all paradigms.

Although many studies have assessed the threshold for perception of linear and angular acceleration^11,26,35,36^, fewer studies have investigated the perception of translation during linear acceleration. Subjects have reported a sense of translation when rotated around their long-body axis about an Earth horizontal axis (barbeque rotation)^10,37,38^, during centrifugation^33,39^, and during off-vertical axis rotation^40,41^. However, during these studies, the linear stimulus was often interpreted as a combination of tilt and translation. For example, during lateral linear acceleration at 0.5 Hz, half of the subjects reported translation only, the rest reported either hilltop, swing, or translation with tilt at the endpoint^42^. Difficulty distinguishing between tilt and translation and the inconsistencies in perceived motion across these earlier studies were due to the way translation was defined. Past studies were asking the subjects to report whole-body translation, rather than perceived translation of the head. So, subjects could have reported translation, when it was in fact only an artifact of tilt motion about a particular body location.

Our results clearly show that subjects perceived translation of their head when the frequency of motion was 0.15 Hz or higher. Also, the amplitude of perceived head translation increased with the equivalent linear displacement. In agreement with this result, previous studies using linear acceleration have shown that the perception of translation distance varied with speed, i.e. distance was overestimated when going slower and underestimated when moving faster^43^.

In our study, the phase of perceived self-motion during OVAR was fairly constant across frequency and tilt angle. In previous studies, various psychophysical methods have been used to measure the phase of perceived self-motion when subjects were exposed to tilt, translation, or a combination of both. These methods, which included a joystick, a pushbutton, or a bar, have shown consistent phase leads or lags across stimulus frequency. For example, previous studies have reported that OVAR induces a phase lead overall, but no changes across stimulus frequency^27,44^ similar to the present study. Using the large amplitude vertical motion device at NASA Ames, Malcolm and Melvill Jones^45^ found no consistent phase errors in motion perception between 0.1 and 0.5 Hz. Using sinusoidal horizontal linear accelerations other authors found a phase lag in perception of motion that did not change across frequency ranging from 0.0083 to 0.33 Hz^26,27^.

The phase-locking of the joystick tilt responses during OVAR is consistent with near zero phase shifts observed with a “somatosensory” bar technique during a combination of EHAR, VRC and LLT motion paradigms^46^. One of the limitations of our study is the lack of comparable phase responses across all four motion paradigms. Based on this prior data of Merfeld and colleagues [Park 2006]; however, it is reasonable to assume we would have also observed similar near zero phase responses across all frequencies and motion paradigms. While a low-pass filter would predict increasing phase lag at higher frequencies, Holly et al.^47^ previously demonstrated a model based on phase-linking between perceived tilt and translation provided the best fit to the data.

Another limitation of this study is that non-directional cues of motion (vibration and apparatus noise) that are inherently present in each of the motion devices may have contributed to the perceptual responses^48,49^. The periodic nature of the stimulus may have also contributed to the responses. For example, Seidman and colleagues^48^ used transient stimuli or the initial responses of periodic motion and concluded both the linear VOR and translation perception showed high-pass filtering characteristics. This contrasts with other studies that utilized periodic stimuli and concluded that simple filtering does not contribute substantially to human perception of tilt or translation in the same way that eye movements [e.g., Ref.^27^]. Due to the periodic nature of our stimuli, we found that displacement-based perceptual reports, for both verbal and joystick, to be the most intuitive. Mergner et al.^50^ concluded that perceived displacement estimates provide analogous results as perceived velocity estimates during velocity step type stimuli, although other studies using perceived velocity^48^ showed changes in phase across frequencies in contrast to our findings.

### Crossover frequency range for tilt and translation

The motion frequency for which subjects perceived either tilt or perception occurred at < 0.15 Hz for LLT, 0.3 Hz for OVAR and EHAR, and 0.6 Hz for EHAR (Fig. 4). The difference between the motion paradigms might be related to the fact that OVAR and EHAR generated an actual tilt of the body, whereas VRC and LLT only generated an equivalent tilt of the resultant force vector. Also, the magnitude of the resulting gravitoinertial acceleration vector was larger during LLT and VRC than during OVAR and EHAR. This observation is consistent with the hypothesis that both frequency segregation and multi-sensory integration are contributing to resolving tilt-translation ambiguity.

The 0.3 Hz crossover frequency for perception of tilt and perception of translation motion during OVAR has been previously reported^28,51^. These previous studies also suggested a relationship between this crossover frequency and the latency to onset of motion sickness during OVAR. Peak nauseogenicity levels during OVAR occur at the same frequency range where perception of tilt or translation motion is the most ambiguous^28,51–53^. A proposed explanation for motion sickness is that passive movement creates a mismatch between the sensory information related to orientation and movement supplied by the visual and the vestibular systems, and this mismatch induces nausea^54^. In agreement with this theory, motion sickness susceptibility increases during passive motion in the frequency range where the CNS has difficulty distinguishing between tilt and translation.

Traditional assessments of 3-dimensional eye movements during OVAR have determined that the gain of tilt ocular responses, such as the ocular counter rolling (torsion) and counter pitching (vertical), decrease with increasing frequency with increasing phase lag, performing like a low pass filter^28,55^. By contrast, the gain of translation ocular responses, such as the horizontal slow phase velocity and vergence, are characterized as a high-pass filter because their gain increase (and their phase decrease) with increasing frequency. The crossover point between tilt and translation ocular reflexes occurs around 0.3 Hz^40^, similar to the crossover for perception of tilt and translation motion determined in the present study. During LLT, the phase of the horizontal linear vestibulo-ocular reflex always leads the head velocity, and the phase lead is greatest at the lowest frequency^16^. During VRC, the ocular counter rolling is in phase with the interaural linear acceleration in the frequency range of 0.01–0.33 Hz^56^.

Clinical studies have shown that perceptual reports (i.e., reports of neurovestibular symptoms) often don’t match eye movements^57^. The difference between the phase changes for the eye movements as a function of stimulus frequency reported in previous studies, and the absence of phase change for perception of self-motion relative to the stimuli in our study is presumably due to the contribution of other sensory inputs. For example, using a tilt table and a sled mounted on a centrifuge, Mittelstaedt^58^ demonstrated that extra-vestibular gravity receptors are involved in perception and control of body position. Mergner et al.^59^ also showed that proprioceptive input arising from torsional body movements elicited reflexive eye movements that were in agreement with psychophysical responses of self-motion perception. Past studies have shown that the errors and variability associated with aligning the long-body axis with the apparent gravitational vertical increased when somatosensory cues were diminished^17^. For instance, the ability to accurately perceive tilt relative to gravity decreased when padded restraints were used^60^ or the testing platform involved water immersion^61,62^.

Extra-vestibular input significantly contributes to motion perception, as witnessed in subjects with bilateral loss of vestibular function who are able to perceive some amount of tilt by aligning a visual bar with the perceived gravitational horizontal^4^, and who display ocular counter rolling when tilted with respect to gravity^63^. Clark and Graybiel^64^ also showed that perceptions of tilt after prolonged exposure to tilt on a centrifuge were not significantly different between labyrinthine-defective and normal subjects. Bisdorf et al.^65^ concluded that subjects without vestibular function showed mild loss in sensitivity to upright vertical but had normal mean slow phase eye velocity. Therefore, somatosensory alone may provide a reasonable estimate of uprightedness, whereas reliable vestibular input will help increase sensitivity.

### Consequences for spaceflight

Accurate perception of self-motion is important for maintaining the accurate representation of spatial orientation required for postural balance and sensorimotor control during voluntary movements. This perception is critical in environments where visual, proprioceptive, and vestibular inputs are compromised, such as when piloting with low visibility or when landing after spaceflight. In weightlessness, the perception of tilt is absent and the CNS tends to interpret a change in otolith signals as the result of a body translation^10^. This reinterpretation of tilt-translation could account for the decrease in tilt ocular reflexes that occur after spaceflight^66^. A somatosensory feedback could potentially help restore the sense of tilt during low frequency motion^67^. A simple prosthesis made of vibrotactor arrays placed around the waist is currently being tested on astronauts after return from space missions to improve their ability to null-out tilt motion disturbances.

## Supplementary Information

Supplementary Video 1.

Supplementary Information.

## Data Availability

The data that support the findings of this study are available from the corresponding author, G.C., upon reasonable request.
