# Supplementary material for: Effects of motion paradigm on human perception of tilt and translation
Source: Sci Rep. 2022 Jan 26;12:1430. doi: 10.1038/s41598-022-05483-6 (PMC8792002; doi:10.1038/s41598-022-05483-6)
Supplement: Supplementary file 2 — Supplementary Information. [file 41598_2022_5483_MOESM2_ESM.docx]

**Supplemental material**

*1. Representative example of curve fit for joystick phase data:*


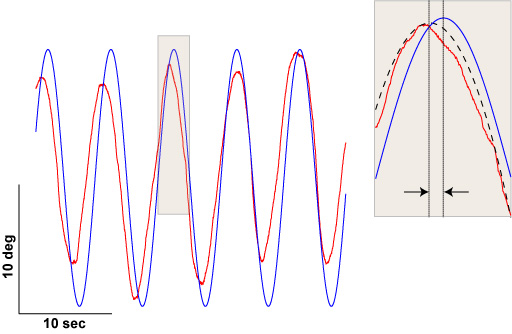


Note the red line represents the joystick data for roll tilt, dashed line (see inset) represents the curve fit, and blue line represents the actual tilt response. Similar data was obtained for the pitch tilt and translation data.

*2. Diagram of perception instructions to the subject:*
